# Hamartin: An Endogenous Neuroprotective Molecule Induced by Hypoxic Preconditioning

**DOI:** 10.3389/fgene.2020.582368

**Published:** 2020-09-30

**Authors:** Sijie Li, Changhong Ren, Christopher Stone, Ankush Chandra, Jiali Xu, Ning Li, Cong Han, Yuchuan Ding, Xunming Ji, Guo Shao

**Affiliations:** ^1^Beijing Key Laboratory of Hypoxic Conditioning Translational Medicine, Xuanwu Hospital, Capital Medical University, Beijing, China; ^2^Inner Mongolia Key Laboratory of Hypoxic Translational Medicine, Baotou Medical College, Baotou, China; ^3^Department of Neurosurgery, Wayne State University School of Medicine, Detroit, MI, United States; ^4^Department of Neurosurgery, The Fifth Medical Centre of PLA General Hospital, Beijing, China; ^5^Public Health Department, Biomedicine Research Center, Basic Medical College, Baotou, China; ^6^Baotou Medical College of Neuroscience Institute, Baotou Medical College, Baotou, China

**Keywords:** hamartin, ischemia, hypoxia, neuroprotection, TSC1

## Abstract

Hypoxic/ischemic preconditioning (HPC/IPC) is an innate neuroprotective mechanism in which a number of endogenous molecules are known to be involved. Tuberous sclerosis complex 1 (TSC1), also known as hamartin, is thought to be one such molecule. It is also known that hamartin is involved as a target in the rapamycin (mTOR) signaling pathway, which functions to integrate a variety of environmental triggers in order to exert control over cellular metabolism and homeostasis. Understanding the role of hamartin in ischemic/hypoxic neuroprotection will provide a novel target for the treatment of hypoxic-ischemic disease. Therefore, the proposed molecular mechanisms of this neuroprotective role and its preconditions are reviewed in this paper, with emphases on the mTOR pathway and the relationship between the expression of hamartin and DNA methylation.

## Introduction

Most aging of the brain is associated with some degree of ischemia/hypoxia for decline in cerebral blood flow (Rosenberg, 2019). Hypoxia/ischemia is a common pathophysiological process seen clinically that can, if left uninterrupted, lead to cell death culminating in serious brain damage (Jovandaric and Milenkovic, 2014; Jiang et al., 2018). Brain ischemia/hypoxia(ischemic stroke) is one of the most common causes of disability and mortality worldwide and is a prominent age-related diseases (Lucke-Wold et al., 2014). Aging is a strong risk factor for poor post-stroke outcome (Roy-O’Reilly et al., 2020). Organisms can forestall this procces, however, using endogenous protective mechanisms that insulate brain cells from the hypoxic/ischemic environment. One of these mechanisms is described by the term hypoxic/ischemic preconditioning (H/IPC), a complex process that appears to function through upregulation of several endogenous molecules that have been shown to exert neuroprotective effects under hypoxic conditions, including VEGF, EPO, and HSP70 (Gora-Kupilas and Josko, 2005; Montero et al., 2007; Bespalov et al., 2014). Hypoxic/ischemic preconditioning is described in detail below.

Tuberous sclerosis complex 1 (TSC1), or hamartin, has recently been proposed as new addition to the list of endogenous neuroprotective molecules (Hadley et al., 2013; Papadakis et al., 2013; Xia et al., 2013). In a study by Papadakis et al. (2013), while hamartin expression was unaffected by hypoxic conditions alone, it was upregulated when preconditioning was performed prior to ischemia, and conferred protection from ischemic injury on the otherwise vulnerable hippocampal CA1 neurons. Hamartin is also known for its capacity to bind with the TSC2 product tuberin, to form a hamartin-tuberin complex that plays a crucial role in the rapamycin (mTOR) signaling pathway (Plank et al., 1998). The mTOR pathway is known to govern cellular responses to hypoxia (Srivastava et al., 2015); therefore, it is not surprising that the neuroprotective function of hamartin was found to proceed through mTOR pathway signaling (Hadley et al., 2019).

The mechanism whereby hamartin expression is unaffected by ischemia alone, and yet is upregulated by preconditioning prior to ischemia, remains unclear. One possible explanation for this phenomenon evokes the role of epigenetics. Studies have shown that a change in DNA methylation at the Tsc1 promoter can affect mRNA and protein expression of mTOR (Zhang et al., 2015), suggesting that epigenetic changes may produce a downstream effect on the neuroprotective function of mTOR. In this article, we review the neuroprotective role of hamartin, provide insight into the role of mTOR pathway signaling in its mechanism, and provide clarity regarding the epigenetic role of DNA methylation in the regulation of hamartin expression.

## Hypoxic/Ischemic Preconditioning (HPC/IPC) and Endogenous Neuroprotection

Hypoxic/ischemic preconditioning (HPC/IPC) refers to a process capable, through prior exposure to a state of moderate hypoxia/ichemia in organisms, organ systems, individual organs, tissues, or cells, of conferring increased resistance to subsequent severe hypoxia/ischemia in these biological units (Shao and Lu, 2012; Altintas et al., 2016). Murry et al. (1986) first described IPC in 1986 after finding that dogs subjected to repeated sublethal ischemia exhibited protection against subsequent sustained cardiac ischemia and reperfusion injury. Although neurons are very sensitive to hypoxia/ischemia, previous research has demonstrated that even tolerance to cerebral ischemia can be induced by IPC (Kitagawa et al., 1990). The underlying mechanisms have not been fully deciphered yet. The process by which this tolerance develops is highly complex, involving a profusion of signaling pathways and their mediators [for example, the Janus-activated kinase (JAK) and PKC], as well as gene expression, together responsible for sensing, transducing, modulating, and effecting preconditioned resistence; these include adenosine, excitatory and inhibitory amino acids (for example, glutamate and γ-amino-butyric acid), reactive oxygen species (for example, O_2_, H_2_O_2_, and OH), transcription factors (for example, NF-kappaB and HIF-1), membrane channels (for example, calcium ions and ATP-sensitive K + channels), heat shock proteins (for example, Hsp-70 and Hsp-27), cytokines (for example, IL-6, IL-1βand TNF-α), and mitochondrial biogenesis (Hagberg et al., 2004; Lu et al., 2005; Long et al., 2006; Marini et al., 2007; Dornbos and Ding, 2012; Thompson et al., 2012; Cai et al., 2014; Chen et al., 2016; Mukandala et al., 2016; Basheer et al., 2018; Jackson et al., 2018). In general, the neuroprotective effect of HPC/IPC appears to depend on both the downregulation of detrimental cellular mediators and biomolecules, and the upregulation of their beneficial counterparts (Lu et al., 2005).

Upregulation of hypoxia inducible factor-1 (HIF-1) by HPC/IPC, for instance, plays a pivotal role in preconditioning-mediated neuroprotection. HIF-1 is a transcription factor responsible for regulating the expression of genes that contribute to hypoxic/ischemic tolerance by modulation, in turn, of several downstream mediators known to be involved in ischemic neuroprotection (Taie et al., 2009). Erythropoietin (EPO) and vascular endothelial growth factor (VEGF) are two of the molecules upregulated by HIF-1, and are known to be stimulants of cell survival and neurogenesis in animal models (Sun et al., 2003; Gu et al., 2008; Chen et al., 2010). EPO can exert neuroprotective effects against hypoxic injury reducing apoptosis by affecting ERK pathways, JAK2/STAT5/Bcl-xL signaling, and others signal transduction pathway (Bartesaghi et al., 2005; Ma et al., 2014; Jeong et al., 2017). VEGF reduced hypoxic lesions in the brain through activation of VEGF signaling, such as VEGF/VEGFR2/Flk1 pathway, MEK/ERK1/2 pathway and so on, to protect neuronal cell from injury (Gomes et al., 2007; Laudenbach et al., 2007). Another mechanism by which HPC/IPC may perform its neuroprotective function is by reducing oxidative damage to tissues and cells. Ischemia/reperfusion injury generates free radicals at concentrations that can damage cellular structures, including proteins, lipids, and DNA (Hatwalne, 2012); HPC/IPC, by contrast, appears to produce these free radicals at a low level that is sufficient to initiate endogenous neuroprotective pathways (Thompson et al., 2012).

Another process that has demonstrated neuroprotective effects in the context of ischemia and may mediate the results of HPC/IPC is DNA methylation, a type of epigenetic modification that regulates gene expression (Hwang et al., 2017). During HPC/IPC, DNA methylation of certain genes is thought to regulate transcriptomic responses to moderate ischemia that ultimately result in the production of ischemic tolerance (Meller et al., 2015). Support for this contention is derived from the finding that DNA methyltransferases (DNMTs), enzymes responsible for DNA methylation, are found to be altered after HPC/IPC. DNMTs can establish specific DNA methylation patterns to protect the brain from damage by modifying gene expression to promote neuroprotection (Zhang et al., 2014; Felling and Song, 2015).

HPC/IPC may also exert its effects on a smaller scale. Modification of protein subunits or amino acids through processes such as phosphorylation alter the activity of the proteins they form and have been shown to be involved in the regulation of several cellular responses in the brain (Takagi, 2014). In support of the importance of phosphorylation to HPC/IPC, Shamloo and Wieloch (1999) found that the level of tyrosine-phosphorylated proteins were increased in the brain after IPC. Similarly, protein phosphatase levels, which regulate dephosphorylation of serine/threonine residues in proteins, were also found to be changed after HPC/IPC treatment (Cid et al., 2007; Zhang et al., 2014). The protein activity regulated by phosphorylation may produce a variety of cellular consequences, including, among others, alteration in the levels of phosphorylated extracellular signal-regulated kinases 1/2 (ERK1/2), change in the location of a protein kinase, and modification of ion influx through the N-methyl-D-aspartate receptor (Shamloo and Wieloch, 1999; Li et al., 2005; Niu et al., 2005; Long et al., 2006; Qi et al., 2007). These changes create a buffer for neurons against hypoxic/ischemic injury caused by autophagy, necroptosis, apoptosis and other mechanisms (Tregub et al., 2016; Ren et al., 2017; Wang et al., 2018).

## The Structure and Activity of Hamartin

**FIGURE 1:**
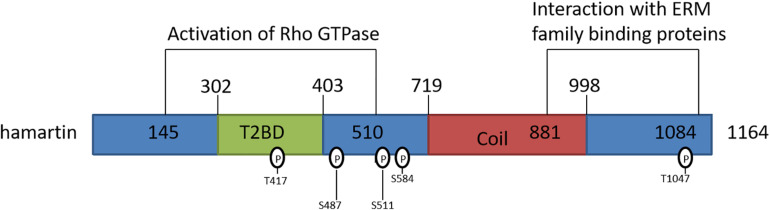
Schematic representation of the hamartin protein and its functional domains. T2BD: TSC2-binding domain; Coil: predicted coiled-coil domain.

Tuberous sclerosis is a disorder involving the formation of hamartomas in multiple organ systems, particularly in the brain, skin, heart, lungs, and kidney (Nguefack et al., 2012; Resende et al., 2013). Studies have identified the TSC1 gene, located on 9q34 (Fryer et al., 1987), as the etiological culprit in this disease. Structurally, the TSC1 gene has 23 exons and produces an 8.6 kb mRNA transcript, the transcriptional product of which is hamartin. Hamartin is a 1,164-amino-acid/130 KDa tumor suppressor protein expressed in most human tissues (Plank et al., 1999; Johnson et al., 2001). It is hydrophilic and has transmembrane domains at amino acids 127–144 and within its coiled-coil region at residues 719–998 (Nellist et al., 1999). Amino acid residues 145–510 contain the functional unit for activation of Rho GTPase, and amino acid residues 881–1,084 interact with the N-terminal domains of the ezrin, radixin, and moesin (ERM) family of actin-binding proteins (Figure 1; van Slegtenhorst et al., 1997; Jacks and Kissil, 2009), which are responsible for motility and neuro-polarization.

The typical molecular activity of hamartin is predicated on the formation of a functional protein complex through binding with tuberin (Slegtenhorst et al., 1998). Although both hamartin and tuberin may have distinct functions outside of their combined complex, hamartin binding to tuberin stablilizes the latter (Chong-Kopera et al., 2006; Huang and Manning, 2008), allowing the complex to proceed to function as the GTPase activating protein (GAP) for the ras homolog RheB, which is highly expressed in the brain (Li et al., 2004). RheB-GTP can interact with the target of rapamycin (TOR) complex 1 (TORC1) to precipitate phosphorylation of TORC1 targets, including p70 S6 kinase and elongation factor 4E binding proteins (Guertin and Sabatini, 2007); thus, formation of the hamartin-tuberin complex is a crucial means by which to inhibit the mTOR pathway.

Differential phosphorylation sites on the hamartin protein may serve as the basis for a “molecular switch” that regulates the formation of its functional complex with tuberin. Astrinidis et al. (2003) demonstrated that endogenous hamartin was threonine-phosphorylated at three sites (Thr 417, Ser 584, and Thr1047) in a reaction catalyzed by cyclin-dependent kinase 1 (CDK1), one of which (Thr417) is located in the hamartin-tuberin interaction domain (Figure 1); the authors proceed to conclude that hamartin phosphorylation controls the activity of the complex during the cell cycle at the G2/M phase. Phosphorylation may also act to negatively regulate the activity of the hamartin-tuberin complex. A study by Lee et al. (2007) suggested that the IKKβ kinase phosphorylated hamartin at both Ser487 (a non-traditional phosphorylation site) and Ser511 (an orthodox phosphorylation site), and found that phosphorylation at these sites enhances dissociation of the complex, which in turn induces mTOR activation.

## The Neuroprotective Role of the Hamartin/mTOR Pathway

The mTOR pathway is critically involved in intracellular signaling events during I/R injury and increases the phosphorylation of the mTOR confers neuroprotection against I/R (Arabian et al., 2019). mTOR has been proposed as a novel target for neuroprotective treatment of hypoxia/ischemia brain injury (Chen et al., 2012). The mTOR pathway modulated autophagy, inducible nitric oxide synthase (iNOS), oxidative state, the mitochondrial and non-mitochondrial oxygen consumption rate, and so on to prevent neurons form hypoxia/ischemia injury (Dutta et al., 2015; Arabian et al., 2019; Zhang et al., 2019).

**FIGURE 2:**
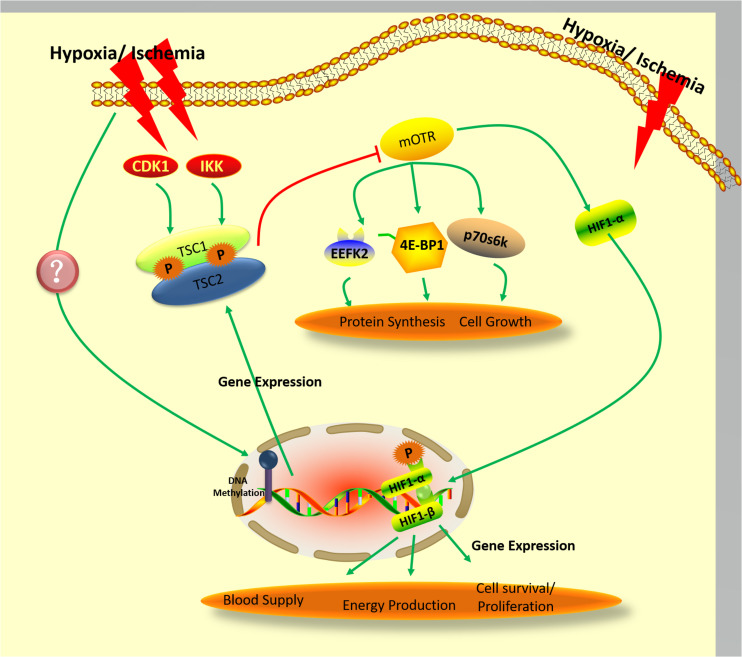
Proposed pathway governing the expression and activity of hamartin under hypoxic/ischemic conditions.

Despite the fact that the full scope of hamartin-tuberin complex function has not been revealed, its role in inhibition of mTOR activity is well-established (Figure 2; Chen et al., 2012). Inactivating variant in either hamartin or tuberin resulted in the hyperactivation of the mechanistic target of mTOR pathway and dysregulated mTOR signaling resulted in increased cell growth and proliferation (Salussolia et al., 2019). It is also clear that important neuroprotective role of TSC in the context of hypoxic/ischemic conditions may depend on mTOR pathway (Liu et al., 2019).

Controversy remains regarding the nature of the alterations to mTOR produced by hypoxic/ischemic conditions, however. Zare Mehrjerdi et al. (2013) found that remote ischemic preconditioning (RIPC) decreased apoptosis, an effect that was associated with increased p-mTOR, while mTOR remained unaltered; mechanistic confirmation was obtained in this study when rapamycin abolished all protective effects of RIPC. On the contrary, Yang et al. (2015) demonstrated essentially the opposite findings: cerebral ischemia in rats resulted in an increase of mTOR transcripts and protein concurrent with apoptotic and necrotic neuronal death, while inhibition of mTOR by rapamycin markedly reduced ischemia-induced damage. Clinical findings have mimicked this latter pattern, with patients treated using rapamycin showing a decrease in the number of stroke or transient ischemic attacks compared with the non-rapamycin control group (Beek et al., 2009). As in the treatment of ischemia, there is also controversy over mTOR activation-mediated modulation of neuroprotection during hypoxia treatment (Bilali et al., 2008; Liu et al., 2015). Chen et al. (2012) address this with their proposal that multiple processes underwritten by mTOR signaling, such as anti-apoptosis, regeneration of neurons, removal of neurotoxins, and angiogenesis, may support neuronal survival in the wake of hypoxic/ischemic brain injury. While, it is worth noting that the clinical use of temsirolimus (CCI-779), an mTOR inhibitor, in oncology (Galanis et al., 2005). It would be of great clinical value to explore positive or negative effects of mTOR inhibitor on neuroprotection in patients with cancer and stroke.

Despite these uncertainties surrounding the pathway’s details, mTOR signaling during ischemia/hypoxia is known to depend on hamartin-tuberin complex formation (Brugarolas et al., 2004; Figure 2). It is also well-established that phosphorylation of tuberin is a crucial step in oxygen-sensing pathways relevant to the cellular response to energy depletion (Inoki et al., 2003; Leontieva and Blagosklonny, 2012). In addition to precipitating cellular energy depletion, hypoxia results in activation of the AMPK/TSC2/RheB pathway, which culminates in mTOR inhibition (Liu et al., 2006). The hamartin-tuberin complex inhibits mTORC1 by acting on RheB when the cell is subjected to hypoxic or energy-poor conditions, and thereby enacts downstream control over protein synthesis and cell growth through regulation of p70S6K, 4E-BP1, and EEF2K (Browne and Proud, 2004). As mentioned above, phosphorylation of hamartin appears to be an important component of the control of hamartin-tuberin complex formation (Astrinidis et al., 2003; Lee et al., 2007), but it is still unclear whether the phosphorylation of hamartin is also involved in oxygen-sensing pathways. Recently, it was reported that protein kinase B (also known as AKT) can regulate IKKβ kinase under ischemic/hypoxic conditions (Chong et al., 2005; Song et al., 2005), and that inhibition of cyclin-dependent kinases (CDK) improves the survival of hippocampal CA1 neurons. AKT regulates the dissociation, while CDK1 regulates the formation, of the hamartin-tuberin complex – either indirectly, or directly through phosphorylation of hamartin (Astrinidis et al., 2003; Lee et al., 2007), as was also mentioned above. It has further been shown that hypoxia can affect the activity of AKT and CDK activity (Kook et al., 2008; Song et al., 2017). Therefore, other signals such as hamartin phosphorylation may also transudce hypoxia, resulting in mTOR inhibition-mediated neuroprotection.

## Hamartin-Mediated Endogenous Neuroprotection

One component of brain physiology that has been particularly useful to efforts to elucidate the role of hamartin in endogenous neuroprotection is the differential resistance to hypoxia exhibited between hippocampal regions. It has been long-established that CA1 hippocampal neurons are highly vulnerable to hypoxic conditions, while CA3 cells are relatively resistant to ischemic injury. This contrast has spurred research interest in determining the molecular foundations of CA3 resistance (Chen et al., 1996; Ouyang et al., 2007; Sun et al., 2009). Hadley et al. (2013), for instance, have investigated hamartin in this context, reporting that, while hamartin levels in CA1 neurons are unaffected during ischemia alone, they are upregulated when antecedent ischemic preconditioning is instituted; by contrast, hamartin can be induced by ischemia in CA3 neurons. This led them to propose that hamartin is a critical mediator both of the resistance of CA3 neurons to global ischemia, and of the tolerance conferred by IPC on CA1 neurons. In addition, knockdown and overexpression studies of hamartin have demonstrated increased and decreased vulnerability of neurons, respectively, to cell death following oxygen-glucose deprivation (Johnson et al., 2001). These findings are consistent with the classification of hamartin as an endogenous neuroprotective molecule in the brain.

The mechanism by which hamartin fulfills its neuroprotective function may involve modulation of metabolic programming on the molecular level:

(1) Hamartin highly related with ATP product and biosynthesis. Wang et al. found increases in mitochondrial respiration, glycolysis, and lipid synthesis in hamartin-deficient dendritic cells (Wang et al., 2013). *TSC1/2^–/–^* cells are hypersensitive to glucose deprivation and this has been linked to increased p53 translation and activation of apoptosis (Choo et al., 2011). These observations appear to translate to larger units of organization, as it was found in another study both that cells containing mutated hamartin were enlarged by a factor of 2–3, and that the size of organs that contained the most hamartin mutant cells were increased (Potter et al., 2001). On the other hand, it has been shown that energy efficiency promotes a reduction in cell size (Sengupta et al., 2013), as well as protection of neurons from ischemic/hypoxic injury (Shao and Lu, 2012). Thus, the upregulation of hamartin induced by ischemic preconditioning may produce the opposite outcome seen with its inhibition, reducing cellular energy demand and thereby conferring protection on the neurons that express it against ischemic insults.
(2) Hamartin modulated autophagy, a critical regulator of cellular metabolism and homeostasis. Autophagy is well known as a physiological which prolongs cell survival though the recycling of cellular macromolecule to generate energy (Rabinowitz and White, 2010). This process replenishes pools of cellular precursors in response to pressure (Ryter et al., 2013). Autophagy, which is a mechanism for the degradation of cellular components that has come to prominence for its involvement in a number of important diseases (such as obesity, cancer, and neurodegenerative disorders), has been revealed to be critical to the regulation of energy balance in the brain (Choo et al., 2010; Coupe and Bouret, 2012). Autophagy might also participate directly in the degradation of glycogen, lipid and protein to produce ATP to meet celluar demand (Mizushima, 2007; Kovsan et al., 2009; Kim and Lee, 2014). Sheng et al. (2010) showed that activation of autophagy occurred during IPC, provided protection against subsequent permanent focal ischemia, and that induction of autophagy with the mTOR inhibitor rapamycin reproduced the neuroprotective effect seen with IPC. Since hamartin is also induced by IPC and functions through mTOR signaling, the endogenous neuroprotective effect of hamartin may depend on autophagy; indeed, hamartin has been shown to promote autophagy through its inhibitory effect on mTORC1 (Hadley et al., 2013; Papadakis et al., 2013; Xia et al., 2013). In addition to its energy-conserving effect secondary to mTOR inhibition, autophagy also appears to exert its neuroprotective effect through an anti-apoptotic mechanism (Jing et al., 2012).

Thus, taken together, the current data suggest that the endogenous neuroprotection conferred by hamartin may arise both from the energy conservation and anti-apoptosis it promotes, in a manner that can proceed either through autophagy, or independently.

## Modulation of Hamartin Expression by DNA Methylation

One mechanism that may account for the expression pattern exhibited by hamartin is epigenetic induction through changes in DNA methylation. The relationship between epigenetics and hamartin has been demonstrated experimentally, with higher methylation rates seen in the hypothalamic neurons of Sprague Dawley (SD) rats that received high-fat ketogenic diets found to correspond to decreased expression of hamartin (Zhang et al., 2015). Similarly, Wang et al. (2017) revealed reduced expression of hamartin in fibrotic mouse lungs concurrently with an increase in hamartin promoter methylation.

As mentioned earlier, DNA methylation is a type of epigenetic modification that involves potentially stable, heritable genetic modifications that control gene expression, typically without altering DNA sequences (Petronis, 2010). Developmental, environmental, or pathogenic stimuli can cause epigenetic changes, which can affect gene expression and thus the regulation of many cellular processes (Shetty et al., 2018). DNA methylation is the best-studied epigenetic event, and has been found to take place at the 5-C position of the cytosine residues of CpG dinucleotides in a reaction that is catalyzed by DNA methyltransferase (DNMT) (Zhang et al., 2014; Schubeler, 2015). Higher methylation rates of CpG dinucleotides in promoters represses gene expression, while lower methylation rates promote gene expression by facilitating transcription factor binding and the attraction of methyl-binding proteins (Fazzari and Greally, 2004). It has been found that gene expression and DNA methylation changes in aneurysmal subarachnoid hemorrhage patients undergoing remote ischemic preconditioning are involved in coordinated cell cycle and inflammatory responses (Nikkola et al., 2015). IPC induction of Arid5a and Nptx2, modulators of neuronal cell death, were shown to be demethylated in regulatory regions, suggesting the involvement of DNA methylation in IPC-induced neuroprotection (Cai et al., 2019). Thus, it follows that lower methylation rates in its promoter region could result in increased hamartin expression.

In a recent study by our group, we found that hypoxic preconditioning may change expression and activity of the methyltransferase enzymes DNMT3A and DNMT3B (Sheng et al., 2010). It has been reported that decrease of Dnmt1 expression at 4 days post-ischemia may be related to ischemia-induced delayed neuronal death (Lee et al., 2013). In a study involving methyltransferase-mutated mice, Dnmt^S/+^ heterozygotes were shown to be resistant to mild ischemic damage, suggesting that DNMTs adversely impact neuroprotection after ischemia (Endres et al., 2000). Other work has used the nucleotide analog 5-Aza-2’-deoxycytidine (5-aza-cdR) as a DNMT inhibitor to observe the effect of DNA methylation on gene expression (Desjobert et al., 2015; Zhang et al., 2016). Wang et al. (2017) used this strategy to investigate hamartin, demonstrating that 5-aza-cdR significantly upregulated hamartin levels in lung fibroblast cells. Comparable results were achieved on an oral squamous cell line treated with 5-aza-cdR, which in this case produced a significant increase in expression of TSC genes (Chakraborty et al., 2008). 5-Aza-CdR has been approved by FDA for disease treatment through affecting genes directly or indirectly (Yang et al., 2010; Dastjerdi et al., 2014) 0.5-aza-cdR have been used in a clinical setting in myelodysplastic syndrome (Abou Zahr et al., 2015), Therefore, it implied that 5-Aza-CdR may be used as a potential clinical treatment medicine for ischemia/hypoxia brain damage through up-regulation TSC/down-regulation mTOR. Thus, it is conceivable in light of our work on the modulation of DNMTs by hypoxic preconditioning that ischemic/hypoxic conditions may induce DNMTs to alter DNA methylation rates at the hamartin gene, modulating its expression to promote the neuroprotective effect called for under these circumstances (Figure 2).

## Conclusion

Endogenous neuroprotective molecules such as VEGF and HIF-1 are induced by IPC/HPC, whereupon they act to increase neuronal tolerance to hypoxia/ischemia. Therefore, upregulation of these molecules through either chemical or physical (i.e., IPC/HPC) means may prove beneficial in conferring protection against hypoxic/ischemic insults. Hamartin appears to be one such endogenous neuroprotective molecule, which is also well-known for its role in regulating activity of the mTOR pathway that is responsible for controlling cell metabolism and survival. Hamartin regulates formation of the hamartin/tuberin complex that mediates its activity on the mTOR pathway; complex formation may be modulated by differential phosphorylation. Finally, evidence is emerging that epigenetics may play a role in neuroprotection by impacting the expression of hamartin; specifically, DNA methyltransferase changes may result in upregulation of the expression of hamartin in response to hypoxic conditions.

## Author Contributions

SL, XJ, and GS: review conception and design. CR, JX, NL, and CH: literature review. CS, AC, and YD: language modification. All authors contributed to the article and approved the submitted version.

## Conflict of Interest

The authors declare that the research was conducted in the absence of any commercial or financial relationships that could be construed as a potential conflict of interest.
